# Relationship between the Decomposition Process of Coarse Woody Debris and Fungal Community Structure as Detected by High-Throughput Sequencing in a Deciduous Broad-Leaved Forest in Japan

**DOI:** 10.1371/journal.pone.0131510

**Published:** 2015-06-25

**Authors:** Satoshi Yamashita, Hayato Masuya, Shin Abe, Takashi Masaki, Kimiko Okabe

**Affiliations:** 1 Forestry and Forest Products Research Institute, Tsukuba, Ibaraki, Japan; 2 Tohoku Research Center, Forestry and Forest Products Research Institute, Morioka, Iwate, Japan; Universidade Nova de Lisboa, PORTUGAL

## Abstract

We examined the relationship between the community structure of wood-decaying fungi, detected by high-throughput sequencing, and the decomposition rate using 13 years of data from a forest dynamics plot. For molecular analysis and wood density measurements, drill dust samples were collected from logs and stumps of *Fagus* and *Quercus* in the plot. Regression using a negative exponential model between wood density and time since death revealed that the decomposition rate of *Fagus* was greater than that of *Quercus*. The residual between the expected value obtained from the regression curve and the observed wood density was used as a decomposition rate index. Principal component analysis showed that the fungal community compositions of both *Fagus* and *Quercus* changed with time since death. Principal component analysis axis scores were used as an index of fungal community composition. A structural equation model for each wood genus was used to assess the effect of fungal community structure traits on the decomposition rate and how the fungal community structure was determined by the traits of coarse woody debris. Results of the structural equation model suggested that the decomposition rate of *Fagus* was affected by two fungal community composition components: one that was affected by time since death and another that was not affected by the traits of coarse woody debris. In contrast, the decomposition rate of *Quercus* was not affected by coarse woody debris traits or fungal community structure. These findings suggest that, in the case of *Fagus* coarse woody debris, the fungal community structure is related to the decomposition process of its host substrate. Because fungal community structure is affected partly by the decay stage and wood density of its substrate, these factors influence each other. Further research on interactive effects is needed to improve our understanding of the relationship between fungal community structure and the woody debris decomposition process.

## Introduction

In forest ecosystems, more than 90% of primary products flow directly into the decomposition process in the form of detritus [1]. Many biotic factors, such as tree phylogenetic groups, size of trees, and decomposer communities, play an important role in the decomposition process [2, 3]. Variation in these factors among substrates causes marked differences in the decomposition process.

Diverse wood-decaying fungi inhabit woody debris and degrade cellulose, hemicellulose, and lignin more rapidly than do bacteria [4]. Thus, wood-decaying fungi play the most important role in the decomposer community in the decomposition of woody debris [2, 4]. The high diversity of wood-decaying fungi implies a high functional diversity, and thus fungal diversity likely has a positive effect on the decomposition process. Experimental studies on the relationship between fungal diversity and litter and wood decomposition processes revealed positive [5], negative [6, 7], and neutral [8] relationships. These studies indicated that complementary effects accelerate degradation, whereas competitive interactions among diverse fungi impede decomposition activity during the initial stage of decomposition [6, 9]. However, few studies have investigated the relationship between fungal community structure and decomposition rate in the field (*e*.*g*., [10]).

Wood-decaying fungi have traditionally been evaluated by observation of fungal fruiting bodies. However, recent molecular studies have identified a great diversity of fungi among ecosystems, including the phyllosphere [11], forest soil [12, 13], root systems [14, 15], and woody debris [16, 17]. Several studies have revealed that species diversity and species composition determined by an inventory of fruiting bodies, mycelial extraction, or molecular techniques exhibit large differences [17, 18, 19]. Molecular techniques are thought to be more suitable than only the observation of fruiting bodies for detecting wood-inhabiting fungi in dead wood, because these methods may also detect fungal species that are not currently forming fruiting bodies. Therefore, in this study we applied a high-throughput sequencing technique to identify the fungal communities formed within coarse woody debris (CWD).

We investigated the relationship between fungal community structure and the decomposition process by using 13 years of data obtained from a forest dynamics plot. First, we calculated regression curves for the decomposition of two dominant tree genera in a 6-hectare (ha) plot and then evaluated the relative decomposition rates for individual pieces of CWD. We next surveyed fungal community structure in CWD using a high-throughput sequencing technique. Finally, we performed structural equation modeling to reveal the relationships among CWD traits, fungal community structure, and decomposition processes. The decay conditions of CWD determine the fungal community structure, which in turn affects the decomposition process. In this study, we focused on the effect of fungal communities on the decomposition process, rather than on the effect of decay conditions on fungal communities.

## Materials and Methods

### Study site

This study was conducted in a broad-leaved, old-growth forest in Ogawa Forest Reserve (36'56"N, 80 140'35"E; 610–660 m above sea level; permission from the Ibaraki District Forest Office of the Forestry Agency in Japan) in the southern part of the Abukuma Mountains along the Pacific Coast of Japan [20]. The mean annual air temperature and annual precipitation in this forest reserve are 10.7°C and 1910 mm, respectively [21]. Within the 6-ha plot established in this forest, species belonging to Fagaceae are dominant, with *Quercus serrata* as the most predominant species (total basal area: 8.58 m^2^/ha), followed by *Fagus japonica* (6.66 m^2^/ha), *Fagus crenata* (2.77 m^2^/ha), *Castanea crenata* (1.34 m^2^/ha), and *Quercus mongolica* var. *grosseserrata* (1.20 m^2^/ha) [22]. *Carpinus laxiflora*, a member of Betulaceae, has a total basal area of 1.36 m^2^/ha. All individual trees with a diameter at breast height (DBH) ≥ 10 cm in the 6-ha plot were marked by numbered tags, and we recorded the DBH, tree species, location in the plot, and mortality of each individual every 5 years beginning in 1997.

### Characteristics of coarse woody debris

CWD (length ≥ 1 m, DBH ≥ 11 cm), including fallen trunks and snags of *Fagus* and *Quercus*, was searched for in the plot in late September 2012. Individual pieces of CWD were identified using numbered tags. If tags were absent from a piece of CWD, the piece was identified using information on DBH, spatial position in the plot, and tree species.

In mid-October 2012, we drilled to a depth of 10 cm into the side of each piece of *Fagus* and *Quercus* CWD using a drill bit (2 cm in diameter) and collected drill dust samples from five holes to measure the wood density. Samples were collected at the center, at both ends, and midway between the center and each end of the CWD. If the diameter of the CWD at either end was smaller than 10 cm, we collected samples at the point at which the diameter was 11 cm. In the case of a dead tree that was still standing (*i*.*e*., a snag), we collected drill dust from points 20, 60, 100, 140, and 180 cm above the forest floor. Each sample was placed individually in a vinyl bag and transported to the laboratory. For each drill dust sample, we recorded dry weight to the nearest 0.01 g using an electronic balance after oven drying at 70°C for 72 h.

### Fungal flora

We collected another set of drill dust samples for extraction of fungal DNA from 10 holes (1 cm in diameter, 10 cm in depth) in the CWD. The arrangement of the holes was identical to that used for the wood density measurement, but drill dust was obtained from both sides of the CWD. Before drilling, we flame-sterilized the drill bit and the surface of the CWD after spraying with ethanol. All drill dust samples from each piece of CWD were placed in a single sterilized vinyl bag and stored at -20°C until molecular analysis.

### DNA extraction and PCR

After mixing thoroughly, each drill dust sample was frozen in liquid nitrogen and crushed using a mortar and pestle. DNA was extracted from the drill dust sample (300 mg) using the DNeasy Plant Mini Kit (Qiagen). We obtained 48 total DNA samples from the wood. The method of Caporaso et al. [23] was adapted for molecular analysis. In brief, the V9 region of 18S ribosomal DNA was targeted. Genomic DNA samples, at a 1:10 dilution, were PCR-amplified in triplicate reactions using primers tagged for each sample, and each 1-μl DNA sample was added to a 25-μl reaction mixture containing 12.5 μl GoTaq premix (Promega Co. Ltd) and 10 pmol each primer (Illumina_Euk_1391f and Illumina_EukBr) [23]. PCR was initiated by a 4-min denaturation step at 95°C, followed by 35 cycles of 45 s at 94°C, 60 s at 57°C, and 90 s at 72°C, with a final extension for 10 min at 72°C using a GeneAmp 9700 thermal cycler (Perkin-Elmer Applied Biosystems). Amplicons were purified by agarose gel electrophoresis, quantified using a Qubit 2.0 Fluorometer (Life Technologies Japan Co. Ltd.) and pooled at equimolar concentrations into a single volume. The pooled amplicon was sequenced by Illumina Miseq at Frontier Science Research Center, Miyazaki University. The raw sequencing data were deposited as BioProject ID PRJDB3758 on DDBJ Sequence Read Archive (http://trace.ddbj.nig.ac.jp/dra/index.html). The technique described above may also have resulted in detection of inactive fungi.

### Operational taxonomic units

The sequencing data were de-noised and filtered using the QIIME software package version 1.8 [24] (The mapping file required in QIIME was deposited in S1 Table). Our molecular analysis detected not only DNA derived from wood-decaying fungi but also DNA derived from other organisms belonging to Eukaryota. We eliminated eukaryotes other than fungi and included molecular operational taxonomic units (OTUs) belonging to Agaricomycotina, Saccharomycotina, Orbiliomycetes, Leotiomycetes, and Sordariomycetes in the analysis, because they are considered major wood-decaying fungi [4]. We adopted the closed-reference OTU picking process as the OTU picking strategy in QIIME [24]. In this process, reads are clustered against the reference sequence data, and the reads that do not align with sequences in the dataset are excluded. We used datasets comprising small subunit ribosomal RNA sequences from the Silva database release 111 [25], as a reference for each OTU in QIIME. In our preliminary analysis, many reads did not result in a sequence hit within the dataset, but the count of each was fewer than 100 reads. In addition, the phylogenetic position of unassigned reads cannot be determined using phylogenetic analysis, and their function cannot be predicted. Thus, such reads were excluded from the analysis.

### Data

In the dataset, the CWD samples varied in the number of obtained sequencing reads, which resulted in variance in the number of OTUs per CWD sample. To avoid the effect of differences in sequencing depth on the occurrence of OTUs, we applied rarefaction to the dataset using the "rrarefy" function in R. Before rarefying, all OTUs with fewer than 100 reads were excluded from the analysis to prevent inclusion of rare fungal OTUs in the results. The dataset was rarefied to a depth of 1,603 reads (minimum number of reads in the CWD samples; S2 Table). Read counts of each OTU were used as an indicator of abundance. When necessary, the dataset was divided into two groups according to tree genus.

### Analysis

We evaluated the decomposition rate index of each CWD. We assigned a time since death value to each CWD based on forest dynamics data. The year of death of the sampled tree was assigned as the midpoint between the year that tree survival was last confirmed and the year that tree mortality was confirmed. For example, 1999 was chosen as the year of death for trees that died at some point between 1997 and 2001. The time since death was then determined by calculating the difference between the survey year (2012) and the year of death.

The relationship between wood density and time since death was regressed by the following negative exponential model:

D=a×exp(−kt)

where *D* is wood density, *k* is the decomposition constant (year^-1^), *t* is time since death, and *a* is a constant. Then, expected values of wood density for each CWD were estimated using the obtained regression. We constructed separate regression curves for *Fagus* and *Quercus*. Residuals between the expected value of wood density and the observed value were used as an index of the decomposition rate. Large positive index values indicate rapid decomposition, whereas large negative values indicate slow decomposition.

Fungal community structure was evaluated by the number of OTUs, OTU composition, and evenness. The unconstrained ordination method was used to compare fungal OTU composition among CWD samples. Detrended correspondence analysis (DCA) was performed as a preliminary analysis to assess the gradient length for each dataset. The results indicated that the lengths of the *Fagus* and *Quercus* CWD datasets were 2.222 and 1.779, respectively. These results suggest that the response curves could be monotonic, and thus we considered principal component analysis (PCA) to be appropriate. DCA and PCA were performed using PC-ORD version 6 [26]. We used the PCA scores for the three axes (first, second, and third) as variables for the OTU composition of wood-decaying fungi in the following analysis. Number of read counts was log_10_-transformed before PCA analysis. Fungal OTUs with fewer than 10 reads after rarefaction were excluded from the analyses. We used Simpson’s measure of evenness (*E*_1*/d*_) [27, 28] as an index of evenness in the fungal community structure. E_1*/d*_ ranges from 0 to 1 and takes a value of 1 when community composition is completely even.

We used a structural equation model (SEM) to determine how strongly fungal community traits affected the decomposition rate and how the fungal community structure was determined by CWD traits (DBH and time since death). We determined Pearson’s correlation coefficients between CWD and fungal community traits before conducting the SEM analysis to reduce the interactions among variables. If the absolute value of the coefficient was less than 0.2, we omitted the interactions among variables from the SEM analysis. We assumed that CWD traits affected fungal community traits and that both CWD and fungal community traits affected the decomposition rate. We also assumed that fungal community traits potentially vary relative to one another, with the exception of PCA scores. We evaluated the fitness of the model by performing a chi-squared test (*p* > 0.05 indicates a good fit) and by evaluating the comparative fit index (CFI > 0.95 indicates a good fit), the root mean square error of approximation (RMSEA < 0.05 indicates a good fit), and the standardized root mean square residual (SRMSR = 0 indicates a good fit). All variables were *z*-transformed for normalization before analysis. SEM analysis was performed using the “Lavaan” package in the R software for Windows 3.0.2 [29].

A two-way cluster analysis was conducted on the OTUs and the *Fagus* and *Quercus* CWDs to identify groups. We performed the analysis using Euclidean distances and Ward’s linkage method using PC-ORD version 6. The number of read counts was log_10_-transformed prior to analysis.

## Results

### Decomposition process of coarse woody debris

**Fig 1:**
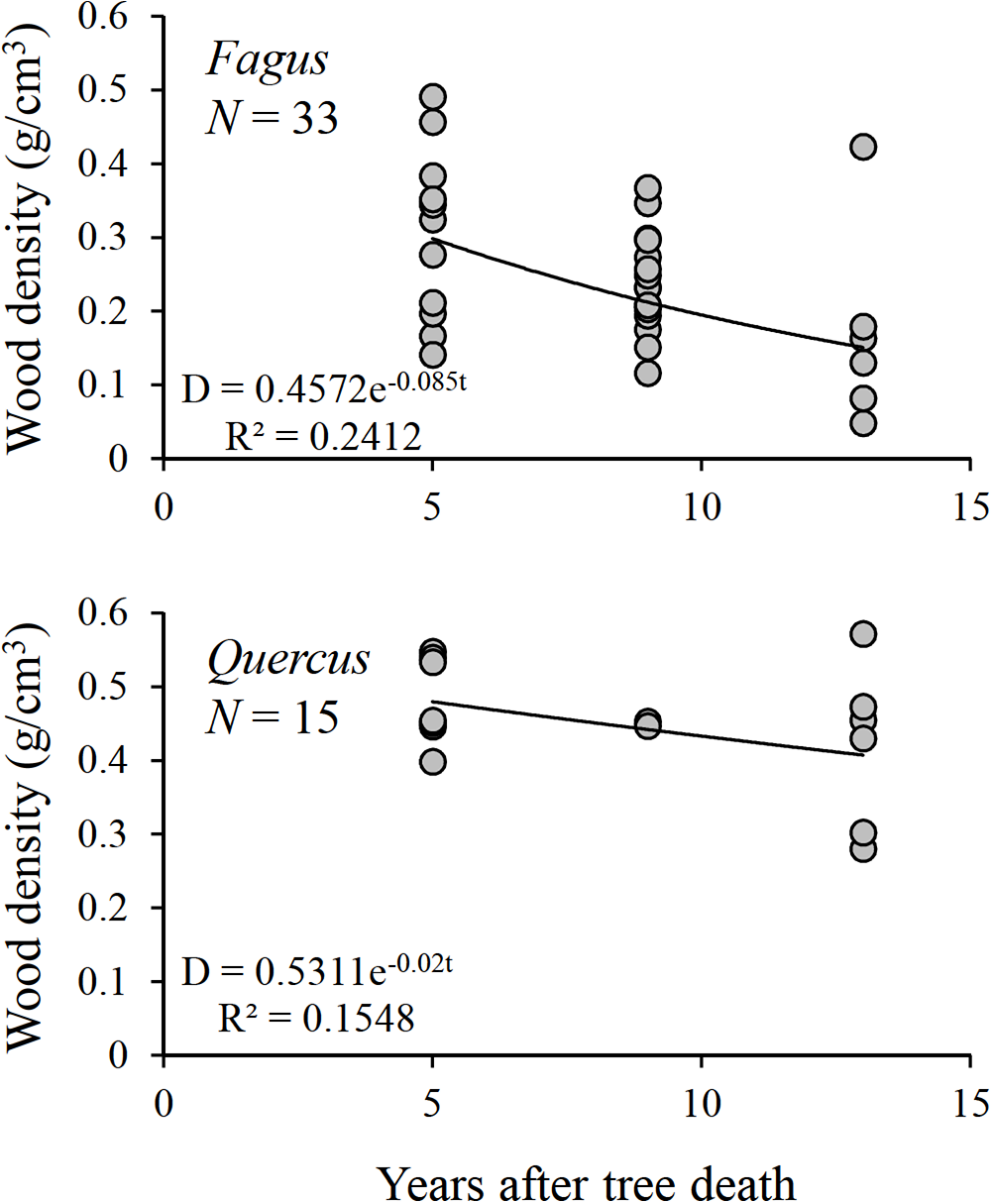
Wood densities of individual pieces of *Fagus* and *Quercus* CWD were plotted against the average time since death. Each wood density value represents the average of five samples collected from each piece of CWD (SD not shown).

In the study plot, 6, 16, and 11 pieces of *Fagus* CWD and 6, 2, and 7 pieces of *Quercus* CWD were obtained between 1997 and 2001, 2001 and 2005, and 2005 and 2009, respectively. The relationship between wood density and time since death was significant for *Fagus* (*D* = 0.4572e^-0.085*t*^, *R*² = 0.2412, *p* = 0.004), but not for *Quercus* (*D* = 0.5311e^-0.020*t*^, *R*² = 0.1548, *p* = 0.147; Fig 1).

### Fungal community

A total of 421 OTUs of Eukaryota from 1,242,841 reads with a length of 250 bp were recorded, and 166 OTUs of fungi from a total of 1,028,993 reads were recorded. For wood-decaying fungi, 120 and 112 OTUs, including singletons, were collected from *Fagus* and *Quercus*, respectively. After excluding those OTUs with fewer than 100 reads, 70 OTUs belonging to wood-decaying fungi were recorded from *Fagus* and *Quercus* each (S2 and S3 Tables).

In *Fagus* CWD, the number of reads ranged from 1,603 to 89,661. *Phallus hadriani* (Agaricales), *Neolentinus lepideus* (Agaricales), *Callistosporium graminicolor* (Agaricales), *Poria cocos* (Agaricales), and *Ceraceomyces borealis* (Agaricales) were the dominant OTUs in *Fagus* (reads > 5%). After rarefication, the number of OTUs ranged from 18 to 48 (S1 Fig), and evenness ranged from 0.048 to 0.142.

In *Quercus* CWD, the numbers of reads ranged from 5,346 to 40,216. *Hericium erinaceum* (Agaricales), Basidiomycete INF1-B (Agaricales), *N*. *lepideus*, *C*. *graminicolor*, *Artomyces pyxidatus* (Agaricales), and *Neobulgaria premonophila* (Leotiales) were the dominant OTUs among *Quercus* (reads > 5%). After rarefication, the number of OTUs ranged from 20 to 44 (S1 Fig), and evenness ranged from 0.045 to 0.261.

OTUs from Agaricomycetes were dominant in both *Fagus* (S2 Fig) and *Quercus* (S3 Fig) CWDs. Of 33 *Fagus* CWDs, 29 were dominated by OTUs from Agaricomycotina, whereas four CWDs were dominated by Leotiales or Saccharomycotina. The relative abundance of the dominant OTUs ranged from 28.2 to 94.5% (58.0± 19.3; average± SD). Of 15 *Quercus* CWDs, 13 and 2 were dominated by Agaricomycotina and Leotiomycetes, respectively. The relative abundance of the dominant OTUs ranged from 23.0 to 94.6% (65.9 20.0, average SD).

**Fig 2:**
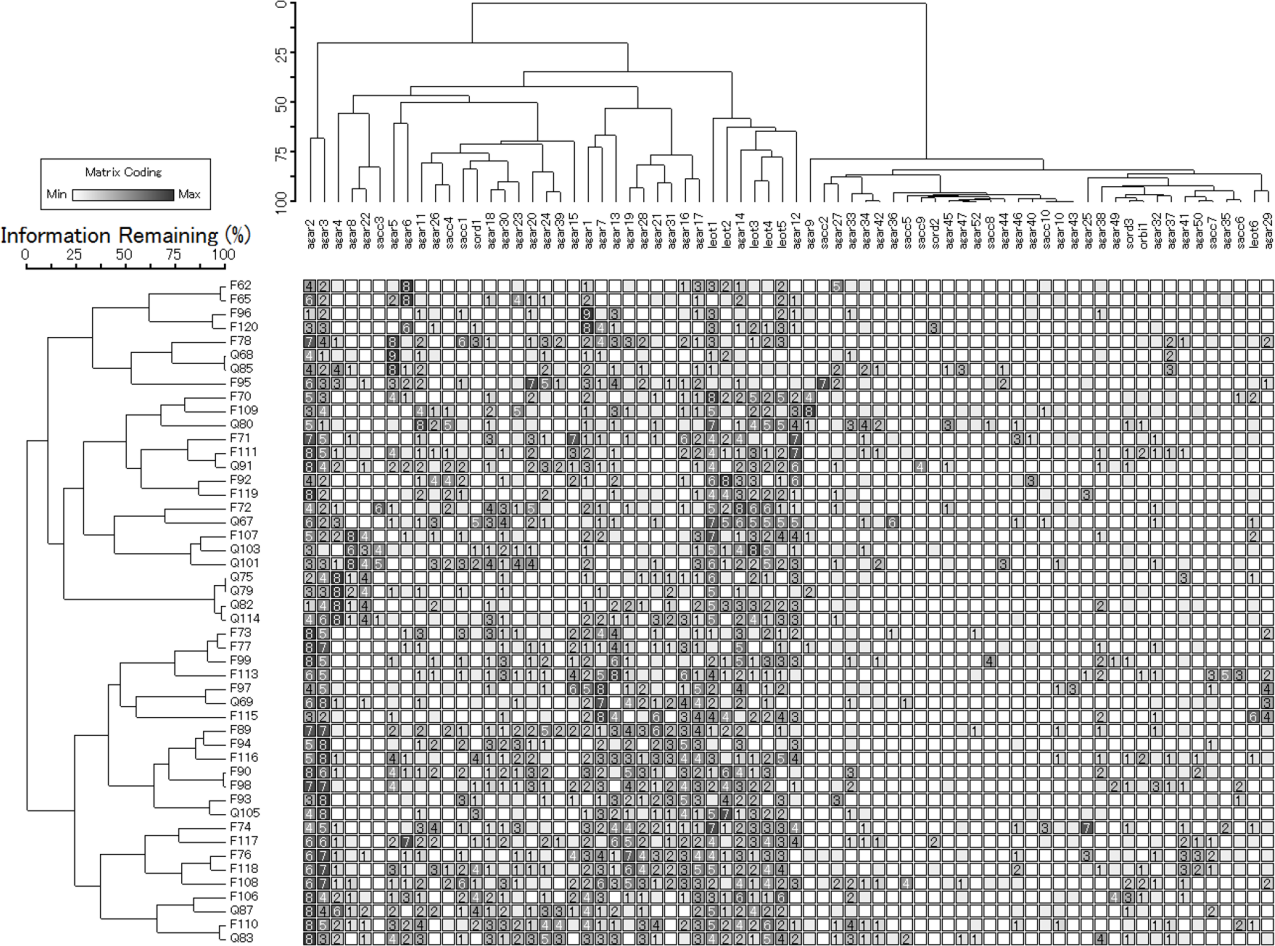
Two-way cluster analysis of fungal OTUs and both *Fagus* and *Quercus* CWDs. Each row represents a CWD, and each column represents an OTU. Columns are clustered according to the abundance of OTUs on CWDs, whereas rows are clustered according to the composition of OTUs. Numbers in the heat map show the matrix coding values, which were relative percent abundance within the matrix.

CWDs were divided into two large clusters according to fungal OTU composition (Fig 2). Both *Fagus* and *Quercus* belonged to each group. Fungal OTUs were also divided into two groups. One group comprised OTUs with high abundance. In this group, *Neolentinus lepideus* (Agar2) and *Callistosporium graminicolor* (Agar3) were observed in almost all CWDs. Five OTUs from Leotiomycetes formed a small cluster in this group. The other group comprised OTUs with low abundance. OTUs belonging to the latter group were observed sporadically.

### Principal component analysis

**Fig 3:**
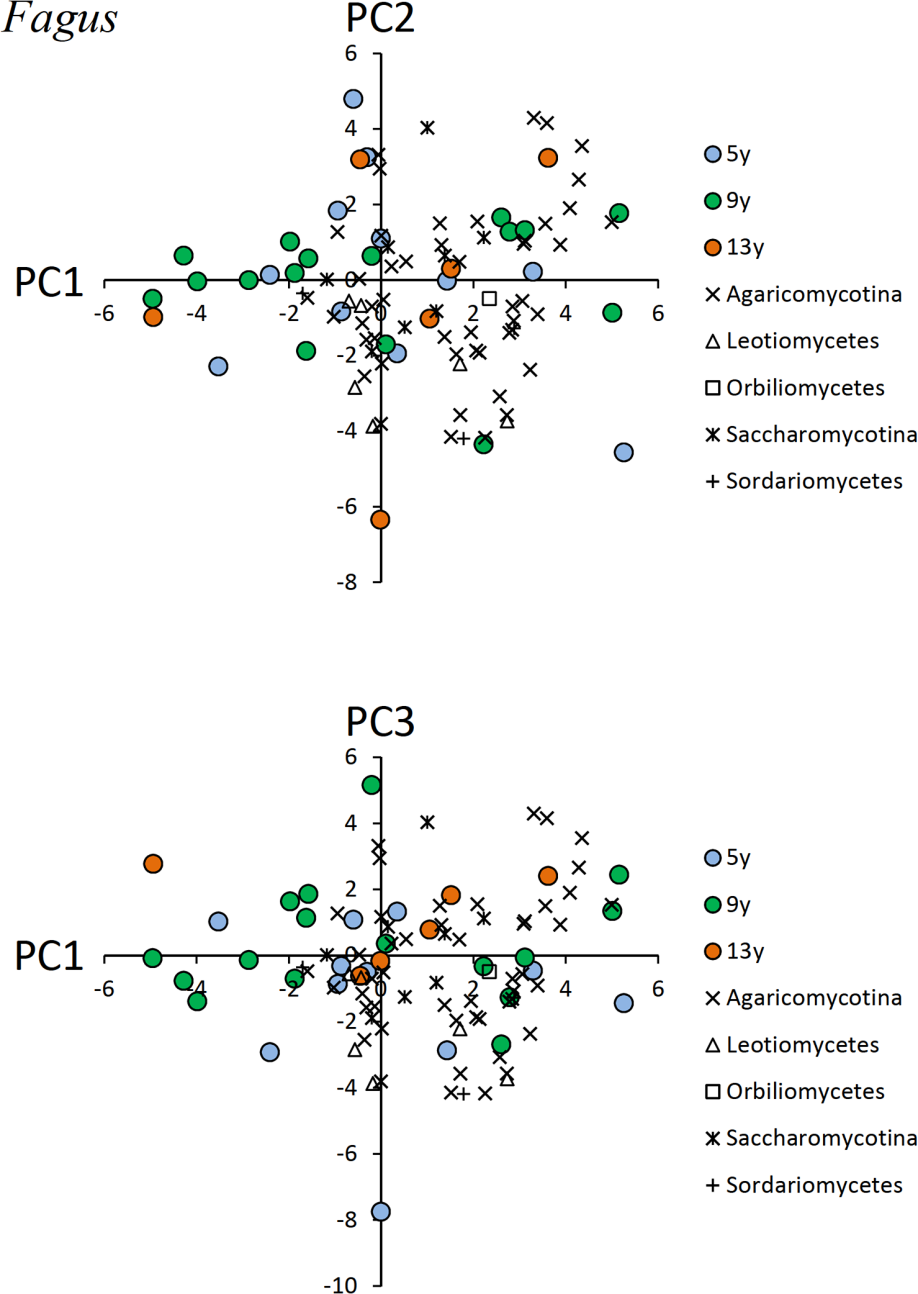
Results of the PCA ordination of individual pieces of *Fagus* CWD and fungal OTUs. Fungal OTUs are presented by subdivision or class to avoid redundancy.

In the PCA results for OTU composition of *Fagus* CWD, the first, second, and third axes explained 12.4%, 8.0%, and 7.4% of the total variance in the OTU data, respectively (Fig 3). In the PCA diagram for PCA scores of the first and third axes, CWDs produced 9 or 13 years ago were plotted in the middle or upper area, respectively, along the PC3 axis, whereas some of the CWDs produced 5 years ago were plotted in the lower area along the PC3 axis.

**Fig 4:**
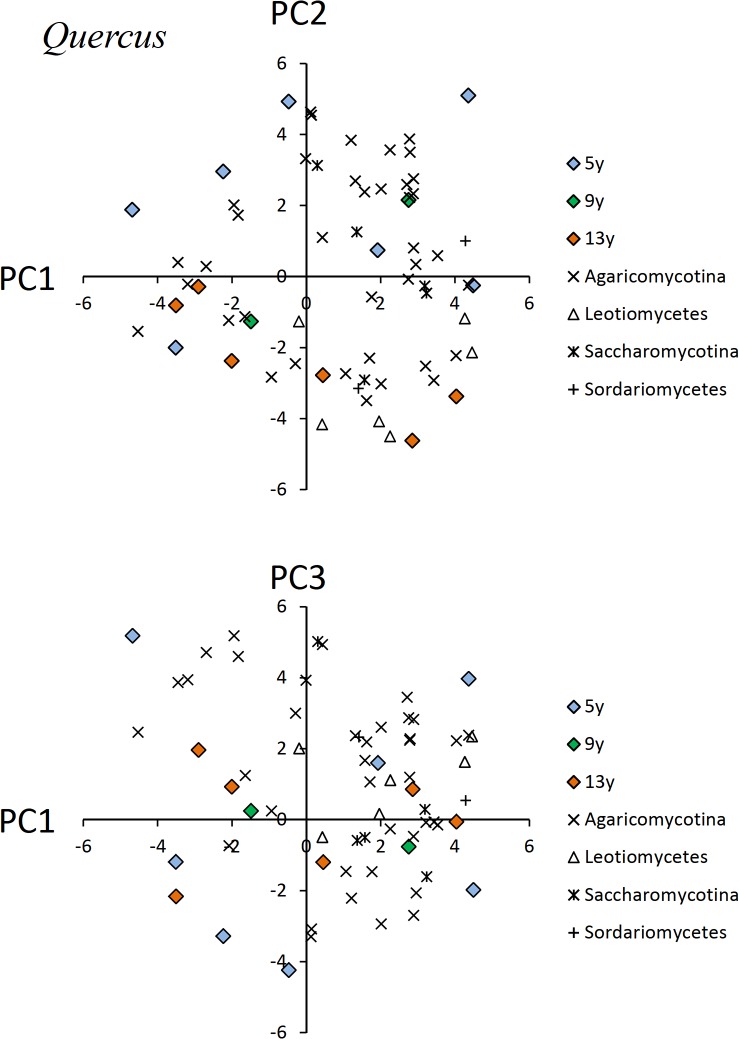
Results of the PCA ordination of individual pieces of *Quercus* CWD and fungal OTUs. Fungal OTUs are presented by subdivision or class to avoid redundancy.

In the PCA results for OTU composition of *Quercus* CWD, the first, second, and third axes explained 19.8%, 16.9%, and 12.8% of the total variance in the OTU data, respectively (Fig 4). In the PCA diagram for PCA scores of the first and second axes, all CWDs produced 13 years ago were plotted on the lower side, whereas five of seven CWDs produced 5 years ago were plotted on the upper side.

### Structural equation model analysis

Pearson’s correlation coefficient was determined before SEM was performed. In *Fagus*, the relationships between DBH and evenness and between time since death and evenness and the PCA score of the third axes showed correlation coefficients greater than 0.2 (Table 1A). All of the fungal community structure traits were correlated moderately with the decomposition rate index (correlation coefficient > 0.2). The number of OTUs and the PCA scores of the second axes were significantly correlated with the decomposition rate index (*p* < 0.05, S4 Fig). CWD traits (DBH and time since death) were poorly correlated with the decomposition rate index (correlation coefficient < 0.2). In *Quercus*, DBH was moderately correlated with evenness and the PCA score of the first and third axes (Table 1B). Time since death was correlated with evenness and the PCA score of the second axis. The decomposition rate was moderately correlated with DBH and evenness. No indices of fungal community structure traits were significantly correlated with the decomposition rate index (*p* > 0.05, S5 Fig).

**Table 1:** Pearson’s correlation coefficients among variables used in the structural equation models.

**(A) *Fagus***	Time	No. OTUs	E_1/*d*_	1st axis	2nd axis	3rd axis	Rate
DBH	0.253	-0.061	0.243	-0.005	0.085	0.035	0.096
Time		-0.007	0.293	-0.013	-0.064	-0.410	-0.068
No. OTUs			0.436	0.896	-0.215	0.219	-0.354
E_1/*d*_				0.458	-0.125	0.044	-0.248
1st axis					0.000	0.000	-0.324
2nd axis						0.000	0.450
3rd axis							0.286
**(B) *Quercus***	Time	No. OTUs	E_1/*d*_	1st axis	2nd axis	3rd axis	Rate
DBH	–0.154	-0.090	-0.260	-0.444	-0.143	0.206	0.409
Time		-0.121	0.330	-0.022	-0.700	-0.008	-0.053
No. OTUs			0.080	0.765	0.105	0.536	0.034
E_1/*d*_				0.184	-0.499	0.245	-0.246
1st axis					0.00	0.00	-0.019
2nd axis						0.00	-0.191
3rd axis							-0.025

Time, time since death; OTUs, operational taxonomic units; E_1/*d*_, index of community evenness; first, second, and third axes, PCA scores of these axes; Rate, decomposition rate index.

**Fig 5:**
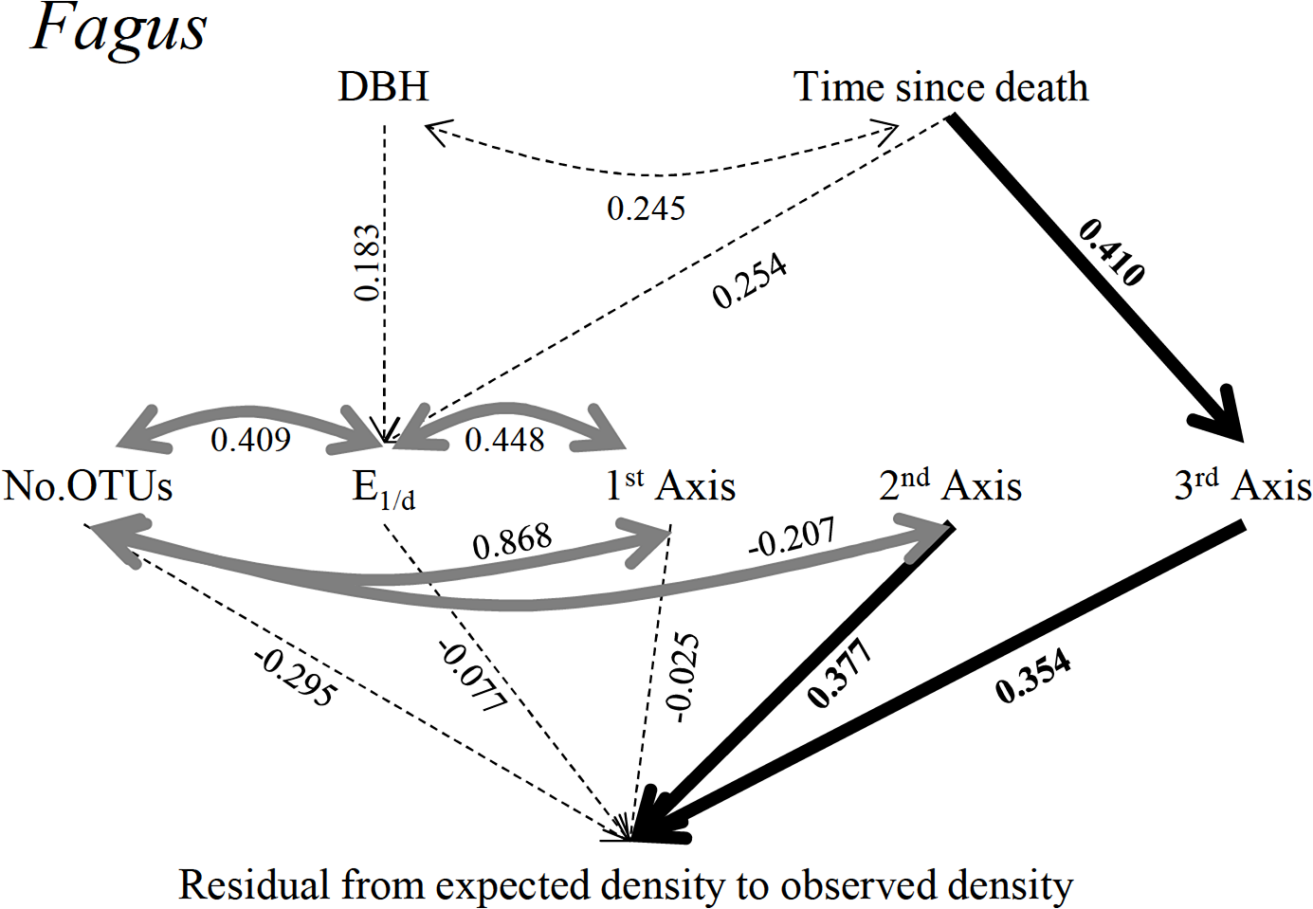
SEM analysis results of the decomposition rate index of *Fagus* CWD. Numbers beside the arrows represent path coefficients. All paths included in the analysis are presented. Straight single-headed arrows represent causal pathways. Curved, double-headed arrows indicate covarying variables. Thick, straight, black arrows indicate significant causal pathways at the level of *p* < 0.05. Thick, gray, curved arrows indicate significant covarying relationships at the level of *p* < 0.05. Dashed, straight, curved arrows indicate non-significance (*p* > 0.05).

SEM for the decomposition process of *Fagus* CWD showed a nearly good fit: the *p*-value by chi-squared test was 0.113, CFI was 0.929, RMSEA was 0.117, and SRMSR was 0.064. In the SEM for *Fagus* (Fig 5), the strongest predictor of the decomposition rate index was the PCA scores of the second axis, with a standardized partial regression coefficient of 0.377 (*p* = 0.014). The standardized partial regression coefficient of PCA scores of the third axis and the decomposition rate index were 0.354 (*p* = 0.009). Time since death significantly affected the PCA scores of the third axis (standard coefficient = 0.410, *p* = 0.010). The number of OTUs varied with evenness and with the PCA scores of the first and second axes. Evenness varied with the PCA scores of the first axis.

**Fig 6:**
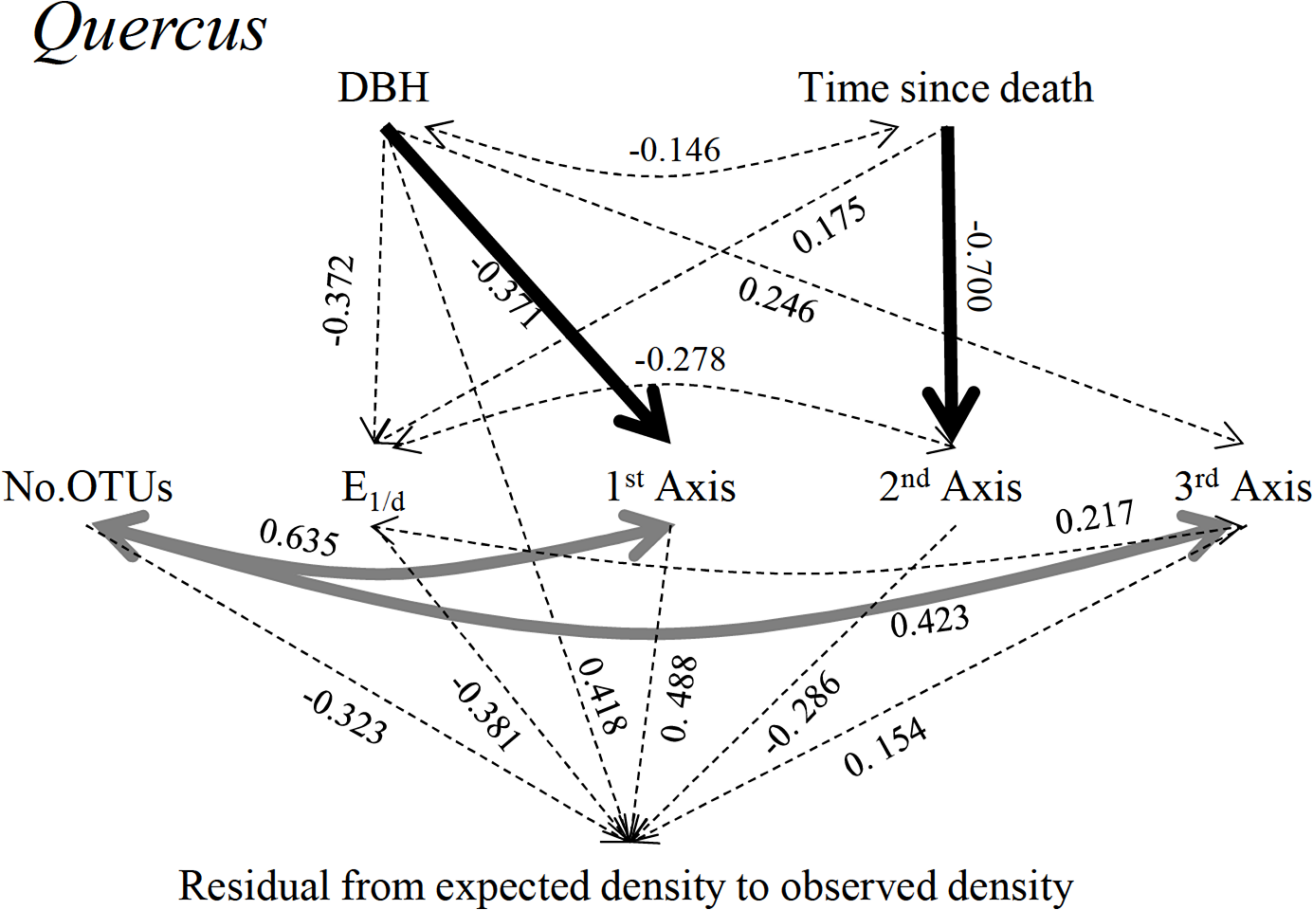
Results of the SEM analysis of the decomposition rate index of *Quercus* CWD. See the legend of Fig 5 for an explanation of the values and arrows.

The SEM results for the decomposition process of *Quercus* CWD showed a good fit: the *p*-value by chi-squared test was 0.916, CFI was 1.000, RMSEA was 0.000, and SRMSR was 0.085. In the SEM for *Quercus* (Fig 6), the strongest predictor of the decomposition rate index was the PCA scores of the first axis, with a standardized partial regression coefficient of 0.488 (*p* = 0.464), although this was not significant at the level of *p* < 0.05. No other factor showed a significant effect on the decomposition rate index. The PCA scores of the first and second axes were significantly affected by DBH (standard coefficient = -0.371, *p* = 0.008) and by the time since death (standard coefficient = -0.700, *p* < 0.001), respectively. The number of OTUs varied significantly with the PCA scores of the first and third axes.

## Discussion

This study focused on the relationships among CWD traits, fungal community structure, and decomposition processes of *Fagus* and *Quercus* CWD. Our analyses revealed three major findings: (1) the decomposition rate of *Fagus* was greater than that of *Quercus*, (2) the fungal community compositions of both *Fagus* and *Quercus* CWD changed over time, and (3) the decomposition rate of *Fagus* CWD was affected by two components of community composition, one that was affected by time since death and another that was not affected by CWD traits but was correlated with the number of OTUs. These findings suggest that fungal community structure is related to the decomposition process of its host substrate, and that the relationship between fungal community structure and decomposition rate is partly affected by CWD traits.

Succession—changes in species composition according to the progression of decay class or time since death of woody debris—has been well described for wood-decaying fungi and related microbes [10, 30, 31]. In a previous study of the fungal community formed on *Fagus* logs, fungal species differed with regard to the substrates that they could decompose efficiently, and fungal succession depended on the chemical traits of the substrate [32]. Thus, it is possible that rapid changes in fungal community composition promote rapid decomposition.

Although many studies have examined the relationships between ecosystem functioning and community structure traits, most have been limited to grassland ecosystems [33] and crop–pollinator systems [34]. With regard to a decomposition system, fungal diversity is thought to affect the decomposition process both positively [5] and negatively [6, 7]. In the SEM analysis, we did not detect a significant relationship between the number of OTUs and decomposition rate, but there was a significant relationship between fungal OTU composition and decomposition rate; moreover, the number of OTUs was significantly correlated with fungal community composition. Therefore, in the case of *Fagus* CWD, the fungal community composition is more strongly related to the decomposition rate than is species diversity.

Fungal community structure for both tree genera were not always explained by the CWD traits. Previous studies showed that the community structure of wood-decaying fungi in a log was affected by wood density, chemical components, moisture content, and log type (*e*.*g*., branch, fallen trunk, stump) [19, 35, 36, 37], and these factors may have determined the community composition at our study site. However, because fungal species appear to disperse randomly at the spatial scale of a few hundred meters [38], the spatial structure of CWD at our small study site may not have had a marked effect on the fungal community structure in this study. Elucidating the interaction between fungi and other saprotrophs, such as bacteria, is also important for understanding the decomposition process. The coexistence of fungi and bacteria stabilizes the decomposition process [39], and commensal interactions between wood-decaying fungi and nitrogen-fixing bacteria are assumed due to their pattern of co-occurrence in CWD [40]. Further studies that include other saprotrophs are needed to understand the relationships between fungal community structure and the decomposition process (see also [41, 42] for wood-inhabiting Myxomycetes and bacteria detected by molecular technique and [43] for interaction between wood-decaying fungi and cryptogam species).

The relationship between fungal community structure and decomposition rate varied with tree genus. In *Quercus*, although the fungal community composition changed over the 13-year period, decomposition did not proceed much during this time. Possibly because of this slow decomposition rate and the small variation in the difference in decomposition rate among CWDs, our SEM analysis did not detect a significant relationship between fungal community structure and the decomposition rate of *Quercus*. This variation among tree genera in the relationship between fungal community structure and the decomposition process requires further research.

The history of community assembly is an important factor in the decomposition process of CWD. Fungi that arrive at a resource unit earlier than others exploit resources and change the chemical composition of CWD during the decay process [44, 45], and they sometimes competitively interact with other fungi that later colonize the resource [46]. Thus, competitive interactions, including the production of secondary metabolites for antagonistic interactions, are important factors in the formation of fungal communities [46, 47]. Perhaps because of such processes, priority effects strongly influence the community structure of wood-decaying fungi and the resulting decomposition process [6, 48]. Such interspecific interactions among fungi also likely affected the assembly of the established fungal communities observed in this study.

We detected 70 OTUs of fungi within CWD of both *Fagus* and *Quercus*. In a previous study on fungal fruiting bodies conducted in the same study forest, 35 and 30 species were recorded in *Fagus* and *Quercus* CWD, respectively [49]. Although the molecular techniques used here revealed high fungal diversity, none of the species detected by observation of fruiting bodies were present in the molecular dataset. Our field survey of fungal fruiting bodies was conducted in 2002 and was limited to aphyllophoraceous fungi, which may explain the discrepancies between these data. In addition, recent studies have reported that the fungal community structure detected by observation of fungal sporocarps is clearly different from that detected by mycelium extracted using a molecular technique [16, 18]. These findings indicate that recently developed methods using high-throughput sequencing fail to detect certain fungal species in CWD.

We used the estimated time since death to illustrate the temporal dynamics of both the decomposition process and fungal community structure. Wood decay stage, instead of time since death, is typically used as an index of CWD age. However, errors in the estimated time since death in this study may be smaller than those based on decay stage. For example, decay stages 3 to 7 in spruce covered CWD from less than 10 years to more than 70 years old [50]. On the other hand, when the focus of the study is the decomposition process, the time since death is usually estimated more accurately than it was in this study. Although our recorded time since death and the actual time since death could differ by as much as 4 years because of our survey schedule, the shape of the curve seemed to be a reasonable expression of the decomposition process. In addition, our decomposition constant for *Fagus* (*k* = 0.085) fell within the range of 0.045 to 0.178 reported for *Fagus sylvatica* [51] and was similar to that reported from Germany (*k* = 0.089) [52]. Likewise, our decomposition rate constant for *Quercus* (0.020) fell within the range of 0.0175 to 0.26 obtained from *Quercus petrea* [51]. In *Quercus*, the loss of outer bark and sapwood occurs within 2 years of tree death [53]. If the same pattern of decomposition is common to both genera, then we underestimated the decomposition rate of CWD in this study. However, our obtained values are not much different from those reported previously.

This is one of the few studies to examine the relationship between fungal community structure and decomposition process under natural conditions. We found that the community composition and evenness of wood-decaying fungi partly determined the decomposition rate of a dominant tree genus, *Fagus*, in a Japanese temperate forest. Because fungal community structure is partly affected by decay stage, wood density and macronutrient composition of its substrate, CWD traits affect the colonization and establishment of the fungal community, which in turn affects the condition of CWD. Evaluations of these interactive effects are necessary to understand the temporal dynamics of the relationships between fungal community structure and the decomposition process.

## Supporting Information

S1 Table Mapping file of the samples. (DOCX)

S2 Table Raw data used in this study. The number of reads was rarefied.
(XLSX)

S3 Table List of OTUs identified from CWD of *Fagus* and *Quercus*. (DOCX)

S1 Fig Frequency distribution of the rarefied numbers of OTUs obtained from CWD of *Fagus* and *Quercus*. (TIF)

S2 Fig Fungal community composition of each *Fagus* CWD at higher taxonomic levels. Abbreviations on each column represent the dominant OTU in each CWD. See S3 Table for the abbreviations.
(TIF)

S3 Fig Fungal community composition in each *Quercus* CWD at higher taxonomic levels. Abbreviations on each column represent the dominant OTU in each CWD. See S3 Table for the abbreviations.
(TIF)

S4 Fig Residual between the expected wood density of *Fagus* CWD and the observed wood density plotted against the indices of fungal community traits formed on each CWD. A large positive value of the residual indicates faster than expected decomposition.
(TIF)

S5 Fig Residual between the expected wood density of a *Quercus* CWD and the observed wood density plotted against the indices of fungal community traits formed on each CWD. A large positive value of the residual indicates faster than expected decomposition.
(TIF)

## References

[1] Cebrian J. Patterns in the fate of production in plant communities. Am Nat. 1999;154: 449–468. 10523491 10.1086/303244

[2] Harmon ME, Franklin JF, Swanson FJ, Sollins P, Gregory SV, Lattin JD, et al Ecology of coarse woody debris in temperate ecosystems. Adv Ecol Res. 1986;15: 133–302.

[3] Zhou L, Dai LM, Gu HY, Zhong L. Review on the decomposition and influence factors of coarse woody debris in forest ecosystem. Journal of Forestry Research. 2007;18: 48–54.

[4] Stokland JN, Siitonen J, Jonsson BG. Biodiversity in dead wood. Cambridge: Cambridge University Press; 2012.

[5] Setälä H, McLean MA. Decomposition rate of organic substrates in relation to the species diversity of soil saprophytic fungi. Oecologia. 2004;139: 98–107. 14740289 10.1007/s00442-003-1478-y

[6] Fukami T, Dickie IA, Wilkie JP, Paulus BC, Park D, Roberts A, et al Assembly history dictates ecosystem functioning: evidence from wood decomposer communities. Ecol Lett. 2010;13: 675–684. 10.1111/j.1461-0248.2010.01465.x 20412280

[7] Peay KG, Dickie IA, Wardle DA, Bellingham PJ, Fukami T. Rat invasion of islands alters fungal community structure, but not wood decomposition rates. Oikos. 2013;122: 258–264.

[8] Dang CK, Chauvet E, Gessner MO. Magnitude and variability of process rates in fungal diversity‐litter decomposition relationships. Ecol Lett. 2005;8: 1129–1137. 10.1111/j.1461-0248.2005.00815.x 21352436

[9] Hättenschwiler S, Tiunov AV, Scheu S. Biodiversity and litter decomposition in terrestrial ecosystems. Annu Rev Evol Syst. 2005;36: 191–218.

[10] Heilmann-Clausen J. A gradient analysis of communities of macrofungi and slime moulds on decaying beech logs. Mycol Res. 2001;105: 575–596.

[11] Jumpponen A, Jones KL. Massively parallel 454 sequencing indicates hyperdiverse fungal communities in temperate *Quercus macrocarpa* phyllosphere. New Phytol. 2009;184: 438–448. 10.1111/j.1469-8137.2009.02990.x 19674337

[12] Buée M, Reich M, Murat C, Morin E, Nilsson RH, Uroz S, et al 454 Pyrosequencing analyses of forest soils reveal an unexpectedly high fungal diversity. New Phytol. 2009;184: 449–456. 10.1111/j.1469-8137.2009.03003.x 19703112

[13] Kadowaki K, Sato H, Yamamoto S, Tanabe AS, Hidaka A, Toju H. Detection of the horizontal spatial structure of soil fungal communities in a natural forest. Popul Ecol. 2014;56: 301–310.

[14] Jumpponen A, Jones KL, Mattox JD, Yaege C. Massively parallel 454‐sequencing of fungal communities in *Quercus* spp. ectomycorrhizas indicates seasonal dynamics in urban and rural sites. Mol Ecol. 2010;19: 41–53. 10.1111/j.1365-294X.2009.04483.x 20331769

[15] Toju H, Sato H, Yamamoto S, Kadowaki K, Tanabe AS, Yazawa S, et al How are plant and fungal communities linked to each other in belowground ecosystems? A massively parallel pyrosequencing analysis of the association specificity of root‐associated fungi and their host plants. Ecol Evol. 2013;3: 3112–3124. 10.1002/ece3.706 24101998 PMC3790555

[16] Allmér J, Vasiliauskas R, Ihrmark K, Stenlid J, Dahlberg A. Wood‐inhabiting fungal communities in woody debris of Norway spruce (*Picea abies* (L.) Karst.), as reflected by sporocarps, mycelial isolations and T‐RFLP identification. FEMS Microbiol Ecol. 2006;55: 57–67. 16420615 10.1111/j.1574-6941.2005.00010.x

[17] Rajala T, Peltoniemi M, Pennanen T, Mäkipää R. Relationship between wood-inhabiting fungi determined by molecular analysis (denaturing gradient gel electrophoresis) and quality of decaying logs. Can J For Res. 2010;40: 2384–2397.

[18] Ovaskainen O, Schigel D, Ali-Kovero H, Auvinen P, Paulin L, Nordén B, et al Combining high-throughput sequencing with fruit body surveys reveals contrasting life-history strategies in fungi. ISME J. 2013;7: 1696–1709. 10.1038/ismej.2013.61 23575372 PMC3749500

[19] Rajala T, Peltoniemi M, Pennanen T, Mäkipää R. Fungal community dynamics in relation to substrate quality of decaying Norway spruce (*Picea abies* [L.] Karst.) logs in boreal forests. FEMS Microbiol Ecol. 2012;81: 494–505. 10.1111/j.1574-6941.2012.01376.x 22458543

[20] Suzuki W. Forest vegetation in and around Ogawa Forest Reserve in relation to human impact In: Nakashizuka T, Matsumoto Y, editors. Diversity and interaction in a temperate forest community: Ogawa Forest Reserve of Japan. Tokyo: Springer-Verlag; 2002 pp. 27–41.

[21] Mizoguchi Y, Morisawa T, Ohtani Y. Climate in Ogawa Forest Reserve In: Nakashizuka T, Matsumoto Y, editors. Diversity and interaction in a temperate forest community: Ogawa Forest Reserve of Japan. Tokyo: Springer-Verlag; 2002 pp. 11–18.

[22] Masaki T, Suzuki W, Niiyama K, Iida S, Tanaka H, Nakashizuka T. Community structure of a species-rich temperate forest, Ogawa Forest Reserve, central Japan. Vegetatio. 1992;98: 97–111.

[23] Caporaso JG, Lauber CL, Walters WA, Berg-Lyons D, Huntley J, Fierer N, et al Ultra-high-throughput microbial community analysis on the Illumina HiSeq and MiSeq platforms. ISME J. 2012;6: 1621–1624. 10.1038/ismej.2012.8 22402401 PMC3400413

[24] Caporaso JG, Kuczynski J, Stombaugh J, Bittinger K, Bushman FD, Costello EK. QIIME allows analysis of high throughput community sequencing data. Nat Methods. 2010;7: 335–336. 10.1038/nmeth.f.303 20383131 PMC3156573

[25] Quast C, Pruesse E, Yilmaz P, Gerken J, Schweer T, Yarza P, et al The SILVA ribosomal RNA gene database project: improved data processing and web-based tools. Nucl Acids Res. 2013;41: D590–D596. 10.1093/nar/gks1219 23193283 PMC3531112

[26] McCune B, Mefford MJ. PC-ORD: multivariate analysis of ecological data. Version 6 Gleneden Beach: MjM Software; 2011.

[27] Smith B, Wilson JB. A consumer's guide to evenness indices. Oikos. 1996;76: 70–82.

[28] Krebs CJ. Ecological methodology. San Francisco: Benjamin/Cummings; 1999.

[29] R Core Team. R: a language and environment for statistical computing. R Foundation for Statistical Computing, Vienna, Austria 2013 http://www.R-project.org/.

[30] Küffer N, Gillet F, Senn-Irlet B, Job D, Aragno M. Ecological determinants of fungal diversity on dead wood in European forests. Fungal Divers. 2008;30: 83–95.

[31] Fukasawa Y, Osono T, Takeda H. Fungal decomposition of woody debris of *Castanopsis sieboldii* in a subtropical old-growth forest. Ecol Res. 2012;27: 211–218.

[32] Fukasawa Y, Osono T, Takeda H. Wood decomposing abilities of diverse lignicolous fungi on nondecayed and decayed beech wood. Mycologia. 2011;103: 474–482. 10.3852/10-246 21262989

[33] Spehn EM, Hector A, Joshi J, Scherer-Lorenzen M, Schmid B, Bazeley-White E, et al Ecosystem effects of biodiversity manipulations in European grasslands. Ecol Monogr. 2005;75: 37–63.

[34] Tscharntke T, Klein AM, Kruess A, Steffan-Dewenter I, Thies C. Landscape perspectives on agricultural intensification and biodiversity–ecosystem service management. Ecol Lett. 2005;8: 857–874.

[35] Heilmann-Clausen J, Christensen M. Wood-inhabiting macrofungi in Danish beech-forests–conflicting diversity patterns and their implications in a conservation perspective. Biol Conserv. 2005;122: 633–642.

[36] Fukasawa Y, Osono T, Takeda H. Microfungus communities of Japanese beech logs at different stages of decay in a cool temperate deciduous forest. Can J For Res. 2009;39: 1606–1614.

[37] Fukasawa Y, Osono T, Takeda H. Dynamics of physicochemical properties and occurrence of fungal fruit bodies during decomposition of coarse woody debris of *Fagus crenata* . J For Res. 2009;14: 20–29.

[38] Yamashita S, Hattori T, Ohkubo T, Nakashizuka T. Spatial distribution of the basidiocarps of aphyllophoraceous fungi in a tropical rainforest on Borneo Island, Malaysia. Mycol Res. 2009;113: 1200–1207. 10.1016/j.mycres.2009.08.004 19682573

[39] Ushio M, Miki T, Balser TC. A coexisting fungal-bacterial community stabilizes soil decomposition activity in a microcosm experiment. PLoS ONE. 2013;8: e80320 10.1371/journal.pone.0080320 24260368 PMC3832447

[40] Hoppe B, Kahl T, Karasch P, Wubet T, Bauhus J, Buscot F, et al Network analysis reveals ecological links between N-fixing bacteria and wood-decaying fungi. PLoS ONE. 2014;9: e88141 10.1371/journal.pone.0088141 24505405 PMC3914916

[41] Clissmann F, Fiore-Donno AM, Hoppe B, Krüger D, Kahl T, Unterseher M, et al. First insight into dead wood protistan diversity: a molecular sampling of bright-spored Myxomycetes (Amoebozoa, slime-moulds) in decaying beech logs. FEMS Microbiol Ecol. 2015: fiv050. 10.1093/femsec/fiv050 25953856

[42] Hoppe B, Krger K, Kahl T, Arnstadt T, Buscot F, Bauhus J, et al. A pyrosequencing insight into sprawling bacterial diversity and community dynamics in decaying deadwood logs of *Fagus sylvatica* and *Picea abies* . Sci Rep. 2015;5: 9456 10.1038/srep09456 25851097 PMC4389208

[43] Fukasawa Y, Takahashi K, Arikawa T, Hattori T, Maekawa N. Fungal wood decomposer activities influence community structures of myxomycetes and bryophytes on coarse woody debris. Fungal Ecol. 2015;14: 44–52.

[44] Kirk TK, Highley TL. Quantitative changes in structural components of conifer woods during decay by white-and brown-rot fungi. Phytopathology. 1973;63: 1338–1342.

[45] Ostrofsky A, Jellison J, Smith KT, Shortle WC. Changes in cation concentrations in red spruce wood decayed by brown rot and white rot fungi. Can J For Res. 1997;27: 567–571.

[46] Boddy L. Interspecific combative interactions between wood‐decaying basidiomycetes. FEMS Microbiol Ecol. 2000;31: 185–194. 10719199 10.1111/j.1574-6941.2000.tb00683.x

[47] Heilmann-Clausen J, Boddy L. Inhibition and stimulation effects in communities of wood decay fungi: exudates from colonized wood influence growth by other species. Microb Ecol. 2005;49: 399–406. 16003479 10.1007/s00248-004-0240-2

[48] Dickie IA, Fukami T, Wilkie JP, Allen RB, Buchanan PK. Do assembly history effects attenuate from species to ecosystem properties? A field test with wood‐inhabiting fungi. Ecol Lett. 2012;15: 133–141. 10.1111/j.1461-0248.2011.01722.x 22188588

[49] Yamashita S, Hattori T, Abe H. Host preference and species richness of wood-inhabiting aphyllophoraceous fungi in a cool temperate area of Japan. Mycologia. 2010;102: 11–19. 20120223 10.3852/09-008

[50] Zielonka T. When does dead wood turn into a substrate for spruce replacement? J Veg Sci. 2006;17: 739–746.

[51] Rock J, Badeck FW, Harmon ME. Estimating decomposition rate constants for European tree species from literature sources. Eur J For Res. 2008;127: 301–313.

[52] Müller-Using S, Bartsch N. Decay dynamic of coarse and fine woody debris of a beech (*Fagus sylvatica* L.) forest in central Germany. Eur J For Res. 2009;128: 287–296.

[53] Schowalter TD, Zhang YL, Sabin TE. Decomposition and nutrient dynamics of oak *Quercus* spp. logs after five years of decomposition. Ecography. 1998;21: 3–10.

