# Supplementary material for: Relationship between the Decomposition Process of Coarse Woody Debris and Fungal Community Structure as Detected by High-Throughput Sequencing in a Deciduous Broad-Leaved Forest in Japan
Source: PLoS One. 2015 Jun 25;10(6):e0131510. doi: 10.1371/journal.pone.0131510 (PMC4481346; doi:10.1371/journal.pone.0131510)
Supplement: S1 Table — (DOCX) [file pone.0131510.s001.docx]

**S1 Table. Mapping file of the samples.**

| CWD | BarcodeSequence | LinkerPrimerSequence | Treatment | ReversePrimer | Description |
| --- | --- | --- | --- | --- | --- |
| F62 | TCCCTTGTCTCC | GTACACACCGCCCGTC | 01_05 | TGATCCTTCTGCAGGTTCACCTAC | Fagus_japonica |
| F65 | TGGTAGTCTGAA | GTACACACCGCCCGTC | 05_09 | TGATCCTTCTGCAGGTTCACCTAC | Fagus_japonica |
| Q67 | CCTATGCACGGT | GTACACACCGCCCGTC | 97_01 | TGATCCTTCTGCAGGTTCACCTAC | Quercus_crispula |
| Q68 | GCGTGGTCATTA | GTACACACCGCCCGTC | 05_09 | TGATCCTTCTGCAGGTTCACCTAC | Quercus_serrata |
| Q69 | AGTCACATCCGC | GTACACACCGCCCGTC | 05_09 | TGATCCTTCTGCAGGTTCACCTAC | Quercus_serrata |
| F70 | AGCGTCTGAACT | GTACACACCGCCCGTC | 01_05 | TGATCCTTCTGCAGGTTCACCTAC | Fagus_japonica |
| F71 | ATCGCGACTGCT | GTACACACCGCCCGTC | 97_01 | TGATCCTTCTGCAGGTTCACCTAC | Fagus_japonica |
| F72 | TGGAGGTTCTCA | GTACACACCGCCCGTC | 97_01 | TGATCCTTCTGCAGGTTCACCTAC | Fagus_japonica |
| F73 | TGCTTGTAGGCA | GTACACACCGCCCGTC | 01_05 | TGATCCTTCTGCAGGTTCACCTAC | Fagus_japonica |
| F74 | CTTAAATGGGCA | GTACACACCGCCCGTC | 01_05 | TGATCCTTCTGCAGGTTCACCTAC | Fagus_japonica |
| Q75 | GGTATCACCCTG | GTACACACCGCCCGTC | 05_09 | TGATCCTTCTGCAGGTTCACCTAC | Quercus_serrata |
| F76 | CGCCTTGATAAG | GTACACACCGCCCGTC | 97_01 | TGATCCTTCTGCAGGTTCACCTAC | Fagus_japonica |
| F77 | CGTTTATCCGTT | GTACACACCGCCCGTC | 05_09 | TGATCCTTCTGCAGGTTCACCTAC | Fagus_japonica |
| F78 | TTGTACTCACTC | GTACACACCGCCCGTC | 05_09 | TGATCCTTCTGCAGGTTCACCTAC | Fagus_japonica |
| Q79 | GCCGCATTCGAT | GTACACACCGCCCGTC | 97_01 | TGATCCTTCTGCAGGTTCACCTAC | Quercus_serrata |
| Q80 | ACGAGACTGATT | GTACACACCGCCCGTC | 05_09 | TGATCCTTCTGCAGGTTCACCTAC | Quercus_serrata |
| Q82 | ATCACCAGGTGT | GTACACACCGCCCGTC | 01_05 | TGATCCTTCTGCAGGTTCACCTAC | Quercus_serrata |
| Q83 | TGGTCAACGATA | GTACACACCGCCCGTC | 05_09 | TGATCCTTCTGCAGGTTCACCTAC | Quercus_serrata |
| Q85 | GTCGTGTAGCCT | GTACACACCGCCCGTC | 05_09 | TGATCCTTCTGCAGGTTCACCTAC | Quercus_crispula |
| Q87 | AGCGGAGGTTAG | GTACACACCGCCCGTC | 05_09 | TGATCCTTCTGCAGGTTCACCTAC | Quercus_serrata |
| F89 | TACAGCGCATAC | GTACACACCGCCCGTC | 01_05 | TGATCCTTCTGCAGGTTCACCTAC | Fagus_japonica |
| F90 | AATTGTGTCGGA | GTACACACCGCCCGTC | 01_05 | TGATCCTTCTGCAGGTTCACCTAC | Fagus_japonica |
| Q91 | TGCATACACTGG | GTACACACCGCCCGTC | 01_05 | TGATCCTTCTGCAGGTTCACCTAC | Quercus_serrata |
| F92 | AGTCGAACGAGG | GTACACACCGCCCGTC | 97_01 | TGATCCTTCTGCAGGTTCACCTAC | Fagus_japonica |
| F93 | ACCAGTGACTCA | GTACACACCGCCCGTC | 05_09 | TGATCCTTCTGCAGGTTCACCTAC | Fagus_japonica |
| F94 | GAATACCAAGTC | GTACACACCGCCCGTC | 97_01 | TGATCCTTCTGCAGGTTCACCTAC | Fagus_japonica |
| F95 | GTAGATCGTGTA | GTACACACCGCCCGTC | 05_09 | TGATCCTTCTGCAGGTTCACCTAC | Fagus_japonica |
| F96 | TAACGTGTGTGC | GTACACACCGCCCGTC | 01_05 | TGATCCTTCTGCAGGTTCACCTAC | Fagus_crenata |
| F97 | CATTATGGCGTG | GTACACACCGCCCGTC | 05_09 | TGATCCTTCTGCAGGTTCACCTAC | Fagus_japonica |
| F98 | CCAATACGCCTG | GTACACACCGCCCGTC | 05_09 | TGATCCTTCTGCAGGTTCACCTAC | Fagus_japonica |
| F99 | GATCTGCGATCC | GTACACACCGCCCGTC | 05_09 | TGATCCTTCTGCAGGTTCACCTAC | Fagus_japonica |
| Q101 | CAGCTCATCAGC | GTACACACCGCCCGTC | 97_01 | TGATCCTTCTGCAGGTTCACCTAC | Quercus_serrata |
| Q103 | GCAACACCATCC | GTACACACCGCCCGTC | 97_01 | TGATCCTTCTGCAGGTTCACCTAC | Quercus_serrata |
| Q105 | CGAGCAATCCTA | GTACACACCGCCCGTC | 97_01 | TGATCCTTCTGCAGGTTCACCTAC | Quercus_serrata |
| F106 | AGTCGTGCACAT | GTACACACCGCCCGTC | 01_05 | TGATCCTTCTGCAGGTTCACCTAC | Fagus_japonica |
| F107 | GTATCTGCGCGT | GTACACACCGCCCGTC | 05_09 | TGATCCTTCTGCAGGTTCACCTAC | Fagus_japonica |
| F108 | CGAGGGAAAGTC | GTACACACCGCCCGTC | 01_05 | TGATCCTTCTGCAGGTTCACCTAC | Fagus_japonica |
| F109 | CAAATTCGGGAT | GTACACACCGCCCGTC | 01_05 | TGATCCTTCTGCAGGTTCACCTAC | Fagus_japonica |
| F110 | AGATTGACCAAC | GTACACACCGCCCGTC | 05_09 | TGATCCTTCTGCAGGTTCACCTAC | Fagus_japonica |
| F111 | AGTTACGAGCTA | GTACACACCGCCCGTC | 01_05 | TGATCCTTCTGCAGGTTCACCTAC | Fagus_japonica |
| F113 | CAACTCCCGTGA | GTACACACCGCCCGTC | 05_09 | TGATCCTTCTGCAGGTTCACCTAC | Fagus_crenata |
| Q114 | TTGCGTTAGCAG | GTACACACCGCCCGTC | 97_01 | TGATCCTTCTGCAGGTTCACCTAC | Quercus_serrata |
| F115 | TACGAGCCCTAA | GTACACACCGCCCGTC | 01_05 | TGATCCTTCTGCAGGTTCACCTAC | Fagus_japonica |
| F116 | CACTACGCTAGA | GTACACACCGCCCGTC | 01_05 | TGATCCTTCTGCAGGTTCACCTAC | Fagus_japonica |
| F117 | TGCAGTCCTCGA | GTACACACCGCCCGTC | 97_01 | TGATCCTTCTGCAGGTTCACCTAC | Fagus_crenata |
| F118 | ACCATAGCTCCG | GTACACACCGCCCGTC | 01_05 | TGATCCTTCTGCAGGTTCACCTAC | Fagus_japonica |
| F119 | TCGACATCTCTT | GTACACACCGCCCGTC | 01_05 | TGATCCTTCTGCAGGTTCACCTAC | Fagus_japonica |
| F120 | GAACACTTTGGA | GTACACACCGCCCGTC | 01_05 | TGATCCTTCTGCAGGTTCACCTAC | Fagus_japonica |
