# Supplementary material for: Relationship between the Decomposition Process of Coarse Woody Debris and Fungal Community Structure as Detected by High-Throughput Sequencing in a Deciduous Broad-Leaved Forest in Japan
Source: PLoS One. 2015 Jun 25;10(6):e0131510. doi: 10.1371/journal.pone.0131510 (PMC4481346; doi:10.1371/journal.pone.0131510)
Supplement: S3 Table — (DOCX) [file pone.0131510.s003.docx]

**S3 Table. List of OTUs identified from CWD of *Fagus* and *Quercus*.**

| Taxa | | | | Abbreviation |
| --- | --- | --- | --- | --- |
| Basidiomycota | | | |  |
|  | Agaricomycotina | | |  |
|  |  |  | Agaricomycetes;__Phallus;__Phallus_hadriani | agar1 |
|  |  |  | Agaricomycetes;__Neolentinus;__Neolentinus_lepideus | agar2 |
|  |  |  | Agaricomycetes;__Callistosporium;__Callistosporium_graminicolor | agar3 |
|  |  |  | Agaricomycetes;__Hericium;__Hericium_erinaceum | agar4 |
|  |  |  | Agaricomycetes;__basidiomycete_INF1-B | agar5 |
|  |  |  | Agaricomycetes;__Poria;__Poria_cocos | agar6 |
|  |  |  | Agaricomycetes;__Ceraceomyces;__Ceraceomyces_borealis | agar7 |
|  |  |  | Agaricomycetes;__Artomyces;__Artomyces_pyxidatus | agar8 |
|  |  |  | Agaricomycetes;__Pulcherricium;__Pulcherricium_caeruleum | agar9 |
|  |  |  | Agaricomycetes;__Serpula;__Serpula_similis | agar10 |
|  |  |  | Agaricomycetes;__Rhizoctonia;__Rhizoctonia_zeae | agar11 |
|  |  |  | Agaricomycetes;__uncultured_eukaryote | agar12 |
|  |  |  | Agaricomycetes;__Clavulina;__Clavulina_sp._MB03-034 | agar13 |
|  |  |  | Agaricomycetes;__Tapinella;__Tapinella_atrotomentosa | agar14 |
|  |  |  | Agaricomycetes;__Pseudohydnum;__Pseudohydnum_gelatinosum | agar15 |
|  |  |  | Agaricomycetes;__Ramariopsis;__Ramariopsis_kunzei | agar16 |
|  |  |  | Agaricomycetes;__Athelia;__Athelia_rolfsii | agar17 |
|  |  |  | Agaricomycetes;__Amanita;__Amanita_bisporigera | agar18 |
|  |  |  | Agaricomycetes;__Agaricus;__Agaricus_bisporus | agar19 |
|  |  |  | Agaricomycetes;__Plicaturopsis;__Plicaturopsis_crispa | agar20 |
|  |  |  | Agaricomycetes;__Volvariella;__Volvariella_gloiocephala | agar21 |
|  |  |  | Agaricomycetes;__Campanella;__Campanella_subdendrophora | agar22 |
|  |  |  | Agaricomycetes;__Amanita;__Amanita_brunnescens | agar23 |
|  |  |  | Agaricomycetes;__Gomphidius;__Gomphidius_roseus | agar24 |
|  |  |  | Agaricomycetes;__Tricholomopsis;__Tricholomopsis_decora | agar25 |
|  |  |  | Agaricomycetes;__Tulasnella;__Tulasnella_violea | agar26 |
|  |  |  | Agaricomycetes;__Lachnocladium;__Lachnocladium_sp._BK171002-23 | agar27 |
|  |  |  | Agaricomycetes;__Hygrocybe;__Hygrocybe_aff._conica_PBM_918 | agar28 |
|  |  |  | Agaricomycetes;__uncultured_Boletaceae | agar29 |
|  |  |  | Agaricomycetes;__Tulasnella;__Tulasnella_asymmetrica | agar30 |
|  |  |  | Agaricomycetes;__Coprinopsis;__Coprinopsis_cinerea | agar31 |
|  |  |  | Agaricomycetes;__Paragyrodon;__Paragyrodon_sphaerosporus | agar32 |
|  |  |  | Calocera;__Calocera_viscosa | agar33 |
|  |  |  | Tremellomycetes;__Tremella;__Tremella_tuckerae | agar34 |
|  |  |  | Agaricomycetes;__Cryptococcus;__Cryptococcus_huempii | agar35 |
|  |  |  | Sebacina;__Sebacina_sp._F1143539 | agar36 |
|  |  |  | Agaricomycetes;__Sparassis;__Sparassis_spathulata | agar37 |
|  |  |  | uncultured_Boletaceae | agar38 |
|  |  |  | Agaricomycetes;__uncultured_marine_eukaryote | agar39 |
|  |  |  | Agaricomycetes;__Henningsomyces;__Henningsomyces_candidus | agar40 |
|  |  |  | Dacrymyces;__Dacrymyces_stillatus | agar41 |
|  |  |  | Tremellomycetes;__Tremella;__Tremella_ramalinae | agar42 |
|  |  |  | Agaricomycetes;__uncultured_Eimeriidae | agar43 |
|  |  |  | Agaricomycetes;__Lactarius;__Lactarius_lignyotus | agar44 |
|  |  |  | Tremellomycetes;__Tremella;__Tremella_foliacea | agar45 |
|  |  |  | Agaricomycetes;__uncultured_Auriculariaceae | agar46 |
|  |  |  | Tremellomycetes;__uncultured_Tremellomycetes | agar47 |
|  |  |  | Agaricomycetes;__uncultured_Boletaceae | agar49 |
|  |  |  | Agaricomycetes;__Ampulloclitocybe;__Ampulloclitocybe_clavipes | agar50 |
|  |  |  | Agaricomycetes;__Multiclavula;__Multiclavula_mucida | agar52 |
| Ascomycota | | | |  |
|  | Pezizomycotina_ | | |  |
|  |  | Leotiomycetes | |  |
|  |  |  | Neobulgaria;__Neobulgaria_premnophila | leot1 |
|  |  |  | Neobulgaria;__Neobulgaria_premnophila | leot2 |
|  |  |  | Neobulgaria;__Neobulgaria_premnophila | leot3 |
|  |  |  | Neobulgaria;__Neobulgaria_premnophila | leot4 |
|  |  |  | Neobulgaria;__Neobulgaria_premnophila | leot5 |
|  |  |  | Neobulgaria;__Neobulgaria_premnophila | leot6 |
|  | Pezizomycotina | | |  |
|  |  | Orbiliomycetes | |  |
|  |  |  | uncultured_Orbiliaceae | orbi1 |
|  | Saccharomycotina | | |  |
|  |  | Saccharomycetes | |  |
|  |  |  | Saccharomycetales;__Anamorphic_Saccharomycetales;__Candida;__Candida_sp._DSM19313 | sacc1 |
|  |  |  | Saccharomycetales;__Pichiaceae;__Yamadazyma;__Candida;__Candida_sp._DSM19311 | sacc2 |
|  |  |  | Saccharomycetales;__Anamorphic_Saccharomycetales;__Candida;__Candida_sp._HA_174 | sacc3 |
|  |  |  | Saccharomycetales;__Anamorphic_Saccharomycetales;__Candida;__Candida_sp._DSM19516 | sacc4 |
|  |  |  | Saccharomycetales;__Anamorphic_Saccharomycetales;__Candida;__Candida_succiphila | sacc5 |
|  |  |  | Saccharomycetales;__Anamorphic_Trichomonascus;__Blastobotrys;__Blastobotrys_terrestris | sacc6 |
|  |  |  | Saccharomycetales;__Anamorphic_Saccharomycetales;__Candida;__Candida_petrohuensis | sacc7 |
|  |  |  | Saccharomycetales;__Anamorphic_Saccharomycetales;__Candida;__Candida_nanaspora | sacc8 |
|  |  |  | Saccharomycetales;__Lipomycetaceae;__Myxozyma;__Myxozyma_monticola | sacc9 |
|  |  |  | Saccharomycetales;__Anamorphic_Saccharomycetales;__Candida;__Candida_santjacobensis | sacc10 |
|  | Pezizomycotina | | |  |
|  |  | Sordariomycetes | |  |
|  |  |  | Phaeoacremonium;__Phaeoacremonium_rubrigenum | sord1 |
|  |  |  | Phialemonium;__Phialemonium_obovatum | sord2 |
|  |  |  | Myrothecium;__Myrothecium_verrucaria | sord3 |
|  |  |  |  |  |
