# Supplementary figures and images for: Relationship between the Decomposition Process of Coarse Woody Debris and Fungal Community Structure as Detected by High-Throughput Sequencing in a Deciduous Broad-Leaved Forest in Japan
Source: PLoS One. 2015 Jun 25;10(6):e0131510. doi: 10.1371/journal.pone.0131510 (PMC4481346; doi:10.1371/journal.pone.0131510)

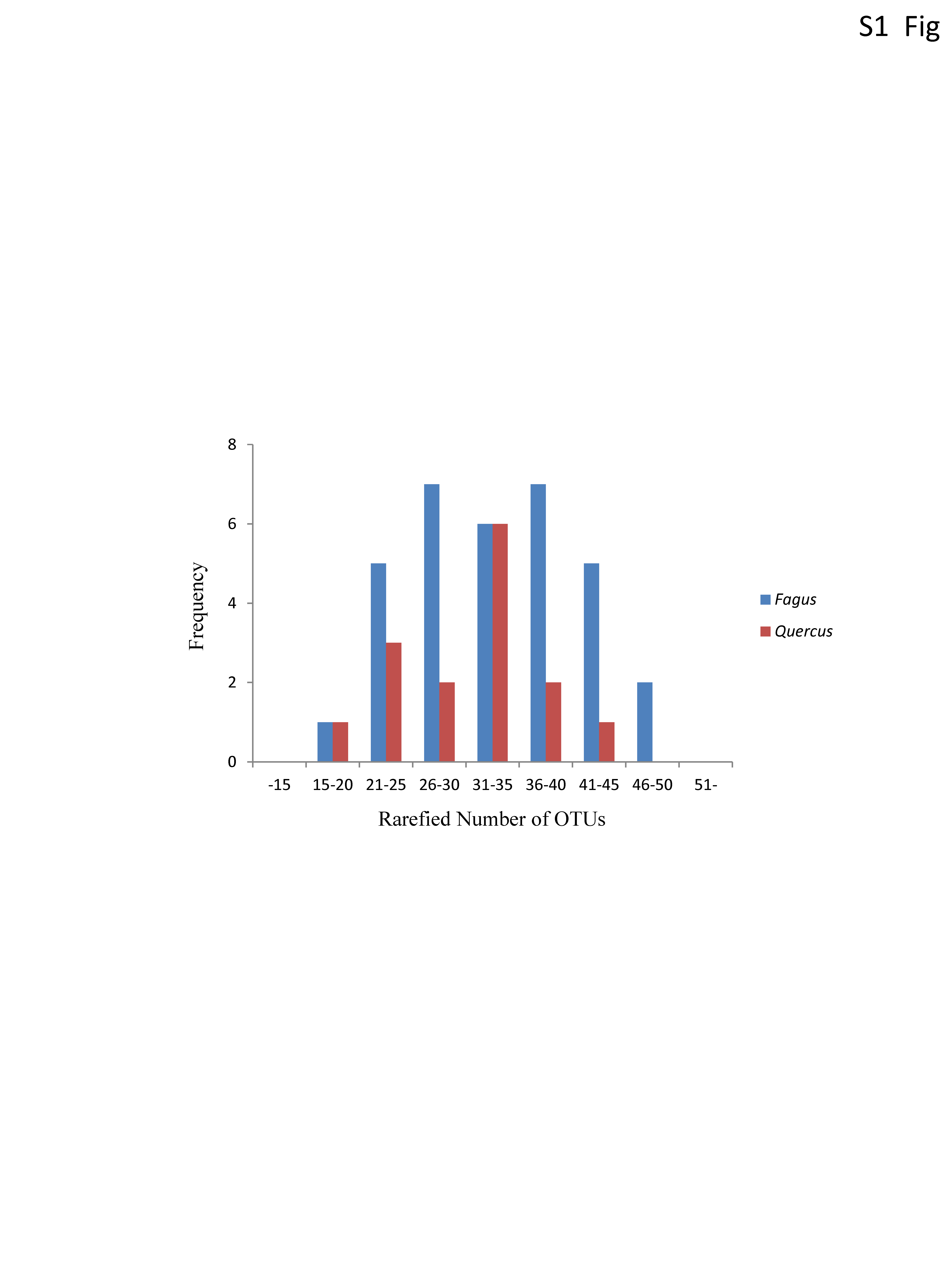

Supplement: S1 Fig — (TIF) [file pone.0131510.s004.tif]

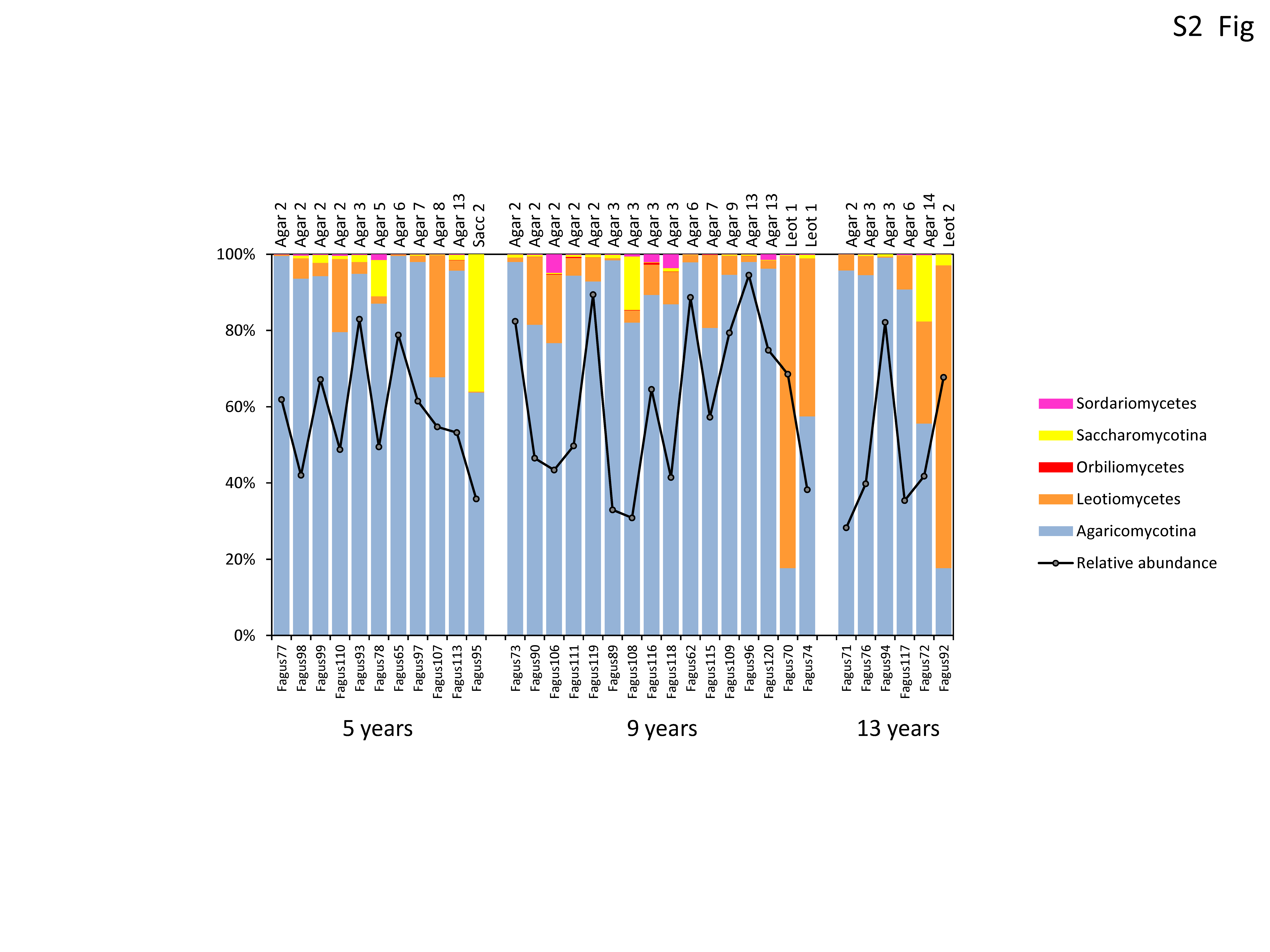

Supplement: S2 Fig — Abbreviations on each column represent the dominant OTU in each CWD. See S3 Table for the abbreviations. (TIF) [file pone.0131510.s005.tif]

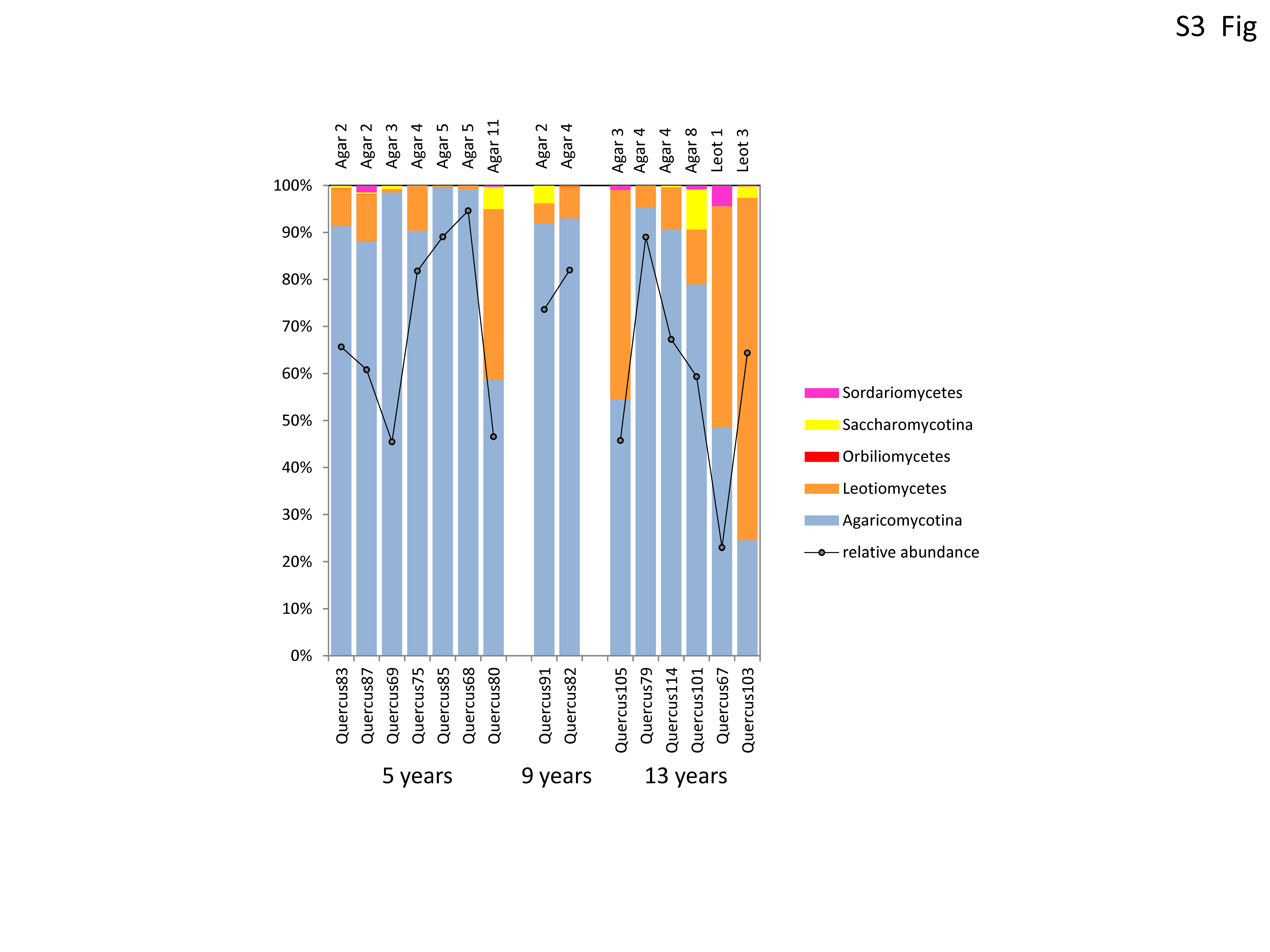

Supplement: S3 Fig — Abbreviations on each column represent the dominant OTU in each CWD. See S3 Table for the abbreviations. (TIF) [file pone.0131510.s006.tif]
